# Unique approaches for testicular shielding during total‐body irradiation for pediatric patients

**DOI:** 10.1002/acm2.13842

**Published:** 2022-11-10

**Authors:** Mohit Shiv Agarwal, Kathryn Bales Kelly, Panos Papanikolaou, Neil Kirby, Chul Soo Ha

**Affiliations:** ^1^ Department of Radiation Oncology, Mays Cancer Center University of Texas Health Science Center at San Antonio San Antonio Texas USA

**Keywords:** gonadal shielding, pediatric, testicular shielding, Total‐body irradiation (TBI)

## Abstract

Total‐body irradiation (TBI) has been used as a part of the conditioning regimen for patients undergoing hematopoietic stem cell transplantation for certain nonmalignant conditions such as sickle cell disease. Although effective, TBI can cause lasting side effects for pediatric patients. One of these potential side effects includes oligospermia or even permanent azoospermia. Although many investigators have studied ways to shield the testicles during the TBI for nonmalignant conditions, there is no set standard. We describe the technical aspects of effective techniques to shield the testicles of male pediatric patients undergoing TBI. We verified that our techniques reduced the testicular dose by approximately 80%–85% of the TBI prescription dose in four male pediatric patients, keeping the dose well below the documented doses that can cause permanent infertility and hypogonadism.

## 1 INTRODUCTION

Total‐body irradiation (TBI) has been an important treatment modality for conditioning regimen prior to hematopoietic stem cell transplantation (HSCT). Recent advances in HSCT have resulted in increased survival for patients undergoing such regimens.^1^ These successes and the growing population of survivors after transplant have placed increasing focus on improving the long‐term sequelae of treatment for these patients.

One of the most common and significant long‐term sequelae of TBI is infertility in men. It is known that the testes are among the most radiosensitive tissues, with relatively low doses causing impairment of spermatogenesis. The addition of cytotoxic chemotherapy, specifically alkylating agents, can also contribute to impaired spermatogenesis.^2^ The mechanism of radiation‐induced infertility in males is complex, but in summary, it is caused by germinal epithelial (Sertoli cell) damage, which results in oligospermia or azoospermia.^3^ This damage can be seen transiently from months to years with radiation dose levels as low as 0.1–1.2 Gy with potential permanent azoospermia seen with fractionated doses over 1.5–2 Gy.^4^, ^5^, ^6^ Additionally, Leydig cells can be damaged, although at significantly higher doses, as they are more radioresistant than the germinal epithelium. Leydig cell damage can result in hypogonadism, which can cause added distress, particularly in pediatric male patients.^7^ One in vitro study showed Leydig cell damage occurring at >6 Gy, whereas other reports cite in vivo data showing damage occurring at >20 Gy.^8^, ^9^, ^10^ Unfortunately, sometimes patients require testicular boosts to cumulative high doses, between 18 and 24 Gy, to reduce the risk of testicular relapse in those with high‐risk disease.^11^ However, in nonmalignant conditions, low dose TBI is used and with reliable gonadal shielding patients should not require testosterone replacement.

There are reports on different strategies for gonadal shielding, including use of the testicular clamshell, a large lead (5 cm^2^ × 8 cm thickness) rectangular block, beam shaping at the collimator, and even implementation of multi‐leaf collimators during volumetric modulated arc therapy‐based TBI.^1^, ^11^, ^12^, ^13^, ^14^, ^15^ We report and depict techniques not previously well described for placing gonadal shielding for patients treated by anterior–posterior/posterior–anterior (AP/PA) fields in an upright, sitting position and also for a patient treated in the lateral decubitus position.

## 2 METHODS

We describe four patients who underwent TBI as part of their conditioning regimens for allogeneic HSCT. We report our testicular shielding technique for these four patients and the optically stimulated luminescence dosimeters (OSLD) results for the gonads from the treatment. All patients had complete history and physical examinations, laboratory, and radiographic evaluations before HSCT.

Patients 1 and 2 were 16‐year old boys with severe sickle cell disease. Both patients initially received an allogeneic matched, related donor transplant with a conditioning regimen of thymoglobulin, thiotepa, fludarabine, cyclophosphamide, and TBI of 200 cGy in one fraction. Both patients experienced a primary graft failure and were planned for a second allogeneic HSCT from a matched, related donor with a conditioning regimen of thymoglobulin, thiotepa, fludarabine, cyclophosphamide, and TBI of 400 cGy in two fractions.

Patient 3 was a 5‐year old boy who suffered from severe sickle cell disease. He was planned to undergo allogeneic HSCT from a matched, related donor with a conditioning regimen of thymoglobulin, thiotepa, fludarabine, cyclophosphamide, and TBI of 200 cGy in one fraction.

Patient 4 was a 3‐year old boy who suffered from transfusion dependent, severe hereditary spherocytosis. He was planned to undergo allogenic HSCT from a matched, related donor with a conditioning regimen of thymoglobulin, thiotepa, fludarabine, cyclophosphamide, and TBI of 400 cGy in two fractions.

**FIGURE 1:**
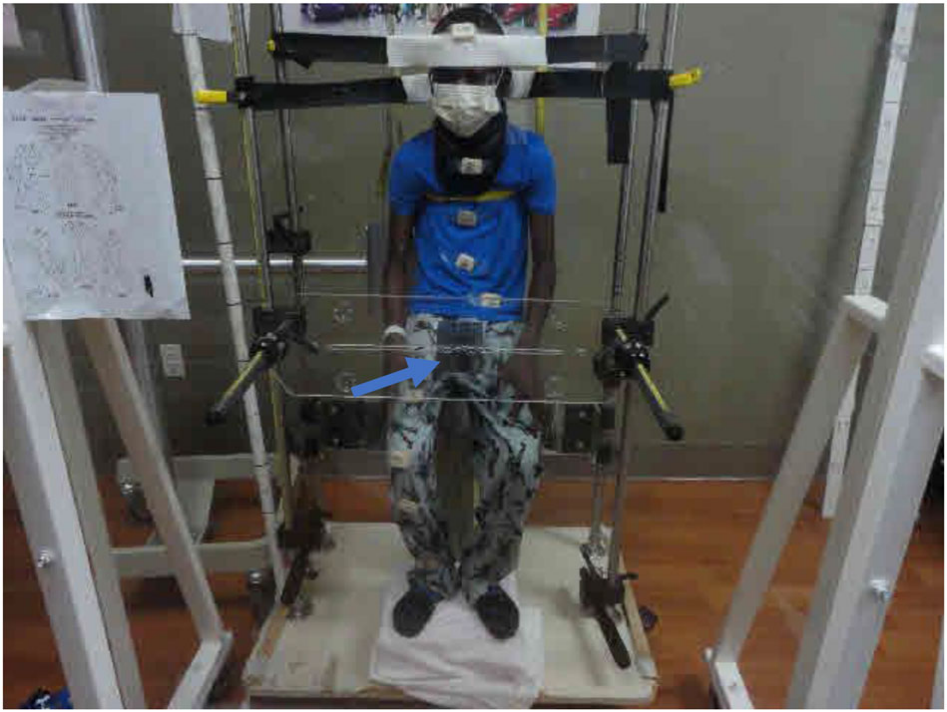
Testicular shielding block (arrow head) screwed into acrylic tray. Block placed by visual inspection of the shadow from the field light

Patients 1 and 2 were positioned in an upright, sitting position on a bicycle seat on a TBI support stand (Figure 1). Patients 3 and 4 were positioned in an acrylic crib in a lateral decubitus position under general anesthesia. All patients were treated using an AP/PA technique at a source‐to‐midplane distance of 340 cm with a 40 × 40 cm^2^ field size to cover the entire body. Customized aluminum compensating filters were constructed for each patient to allow for a relatively uniform dose to be delivered to the patient's midplane. A 1.2 cm thick acrylic spoiler was placed in front of the beam to improve dose homogeneity, and an 8 cm thick lead block (4.7 half‐value layers for 6 MV) was utilized to shield the gonads. This shield was made by stacking four lead blocks that were each 7.6 × 2 × 8 cm^3^ (see Figure 3).

For the setup of the patients in a sitting position (patients 1 and 2), the AP gonadal block was placed by visual inspection of the block shadow from the field light. For the PA orientation, the block was also positioned using the shadow of the field light. Before positioning for PA delivery, the bicycle seat served as a proxy for the inferior extent of the scrotum, that is the lower border of the block shadow was placed about 0.5–1.0 cm below the bicycle seat. After the patient was seated, the blocks were centered on the patient in the left/right direction and the projection of the block shadowed was checked visually from a lateral view of the patient. Portal films were used for both orientations to confirm the gonadal shielding setup.

**FIGURE 2:**
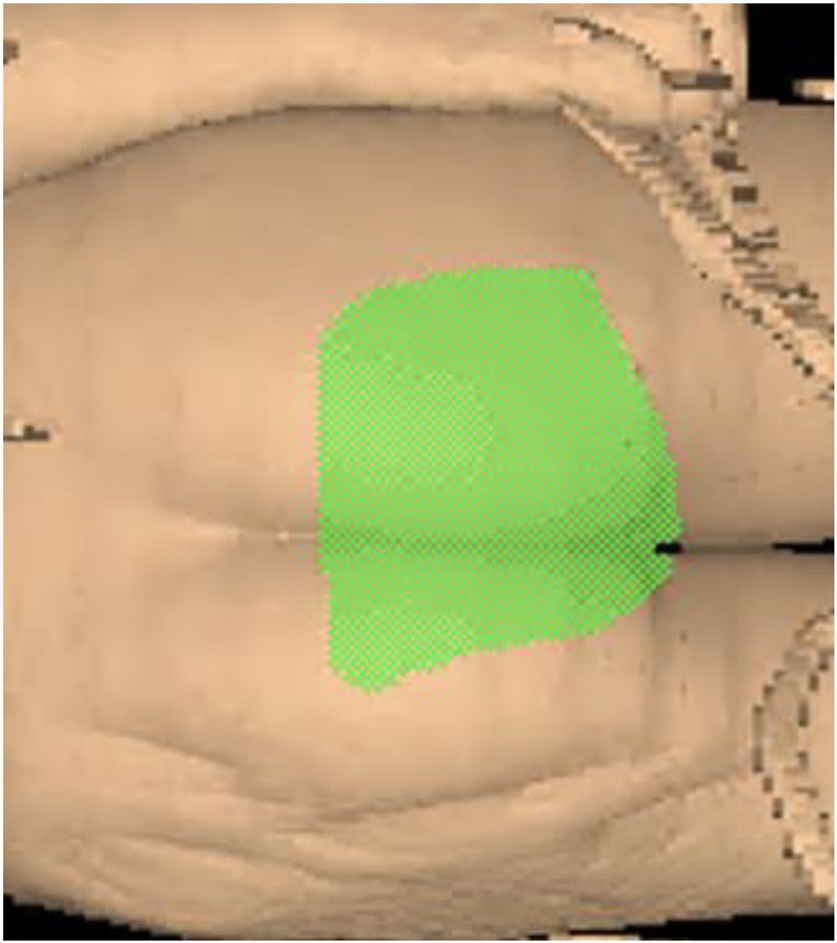
Projection of the posterior–anterior (PA) block on posterior skin surface. Block was contoured to project on desired area to be shielded, which has been defined in the patient's anterior skin surface.

**FIGURE 3:**
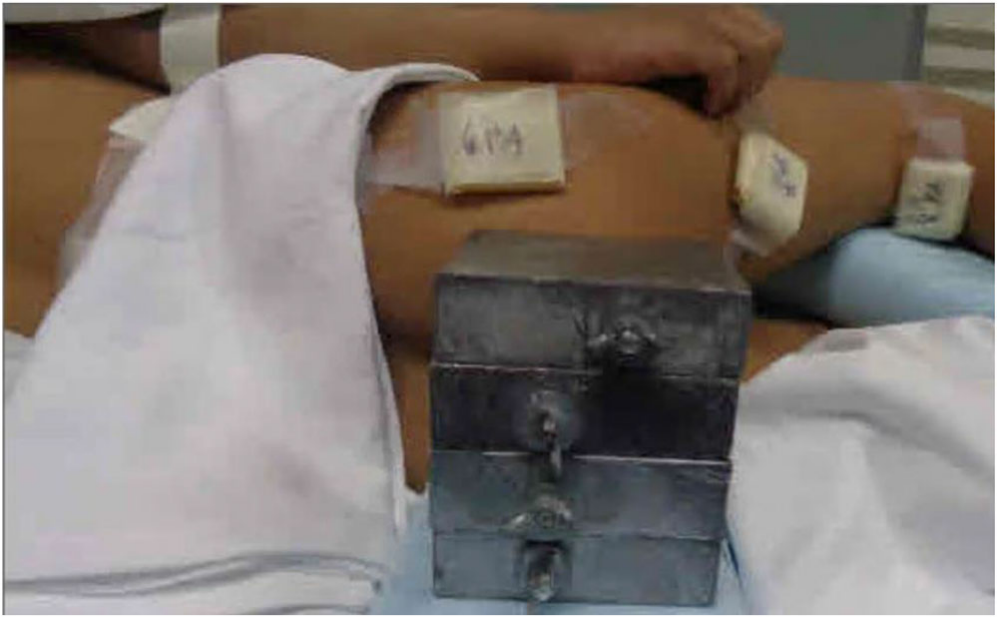
Surface rendering from Figure 2 was used to aid in the positioning of the posterior–anterior (PA) gonadal block during patient's setup.

Because the position of the block required to shield the gonads is difficult to visualize in the PA orientation for the decubitus setup (patients 3 and 4), a wire was placed around the desired area to be shielded in the AP orientation during CT simulation. The patient was scanned, and the resulting image was imported into the Pinnacle treatment planning system. The wire was contoured, and a manual block was created using the wire contour. A PA beam was blocked to this contour and placed using the treatment geometry. The projection of the block on the patients’ posterior skin surface was created (Figure 2). This surface rendering was used to aid in the positioning of the PA gonadal block during patient setup (Figure 3). The AP gonadal block was placed by a visual inspection of the shadow of the block from the field light. Both setups were confirmed using portal films. For each setup, OSLDs, with a one sigma total uncertainity of 4.9%, were placed in the middle of the scrotal surface for both the AP and PA orientations to verify the delivered dose to the testicles.^16^

## 3 RESULTS

The OSLD readings for patient 1 indicated a dose of 43.0 ± 2.1 cGy to the gonads for the first TBI course, a 78.5% decrease from the prescribed dose of 200 cGy. For the second TBI course, this patient received 31.5 ± 1.5 cGy per fraction to the gonads for a total testicular dose of 63.0 cGy, an 84.3% decrease from the prescribed dose of 400 cGy. The OSLD readings for patient 2 indicated a dose of 31.0 ± 1.5 cGy to the gonads for the first TBI course, an 84.5% decrease from the prescribed dose of 200 cGy. For the second TBI course, patient 2 received 37.3 ± 1.8 cGy to the gonads per fraction for a total testicular dose of 74.6 cGy for the TBI treatment, an 81.3% decrease from the prescribed dose of 400 cGy. The OSLD readings for patient 3 indicated a total dose to the testicles of 29.0 ± 1.4 cGy, an 85.5% decrease from the prescribed dose of 200 cGy. The OSLD readings for patient 4 indicated a total dose of 31.0 ± 1.5 cGy to the testicles, an 84.5% decrease from the prescribed dose of 200 cGy.

## 4 DISCUSSION

Testicular shielding is critical in the male pediatric population for TBI treatments in order to preserve future fertility and testicular endocrine function. We describe the techniques for the placement of effective testicular shielding when treating patients using low dose TBI in an upright, sitting position and in the lateral decubitus position. The placement of the gonadal shield is relatively straight forward for the patients treated in an upright, sitting position as we can use the bicycle seat as a proxy for the location of the scrotum. Though the use of TBI in an upright, seated position is conducted at other facilities, we report on methods of more specifics of gonadal shielding using two different techniques. Placement of the shield for the PA beam with the patient in the lateral decubitus position using film dosimetry is time‐consuming, and we propose our technique makes the placement of the PA block much easier by showing where the block shadow should fall under the field light. This eliminates the need for multiple port films. This technique has not been described before in the literature.

## 5 CONCLUSION

In this report, we verify that our newly described gonadal shielding techniques successfully reduced the gonadal dose to about 20% of prescribed TBI dose. The gonadal shielding described in this work is effective and may help reduce the incidence of infertility and hypogonadism in young male low‐dose TBI patients. Therefore, the ongoing research is focused on further reducing the TBI dose by improving the conditioning chemotherapy regimen. Fortunately, however, all four of our patients will have a very good chance of preserving not only the Leydig cell functions but also long‐term spermatogenesis.

## CONFLICTS OF INTEREST

Mohit Shiv Agarwal: conflicts of interest: none.

Kathryn Bales Kelly: conflicts of interest: none.

Panos Papanikolaou: conflicts of interest: none.

Neil Kirby: conflicts of interest: none.

Chul Soo Ha: Protectum Oncology Founder and Chief Medical Officer.

## FUNDING INFORMATION

Mays Cancer Center at UT Health SA: P30 CA054174

## AUTHOR CONTRIBUTION

All authors contributed to the design of the study as well as to the writing and creation of this manuscript.

## Data Availability

All the meaningful data are available in the manuscript.
